# Development of an evidence-based website on preconception health

**DOI:** 10.1080/03009734.2018.1476423

**Published:** 2018-06-18

**Authors:** Maria Ekstrand Ragnar, Jenny Niemeyer Hultstrand, Tanja Tydén, Margareta Larsson

**Affiliations:** Department of Women’s and Children’s Health, Uppsala University, Uppsala, Sweden

**Keywords:** Fertility awareness, health behaviour, internet-based information, preconception health, reproductive life plan

## Abstract

**Introduction:**

Many women and men lack knowledge about fertility, including timing of the fertile window, age-related decline, and lifestyle factors that may impair fertility. The Internet has become an important source of information, but evidence-based information on fertility and reproduction in Swedish on the Internet is limited. The present study aimed to develop and evaluate an evidence-based fertility awareness website, ‘reproduktivlivsplan.se’, to increase awareness of fertility and provide guidance for improved preconception health and care among individuals and healthcare providers.

**Methods:**

The website’s content, design, and layout were evaluated qualitatively among a total of 20 nursing students. An expert group of researchers also provided feedback on the content. Finally, healthcare providers (*n* = 24) answered a questionnaire covering attitudes and views on the Reproductive Life Plan website as a tool for counselling.

**Results:**

The developing process resulted in a mobile-friendly website, ‘reproduktivlivsplan.se’ (in English: Reproductive Life Plan). The website, including the content and layout, was positively evaluated by most participants and was amended according to suggested improvements. Uppsala University was found to be a trustworthy source.

**Conclusion:**

The evidence-based website ‘reproduktivlivsplan.se’ was well received among users and healthcare providers and may provide guidance for improved preconception health and care if it becomes well known and frequently used.

## Introduction

Preconception health and lifestyle factors, such as at what age to have children, nutritional status, weight, tobacco- and alcohol use, are important determinants of fertility, healthy pregnancy, and normal foetal development (1–3). Studies have shown that women and men lack knowledge about fertility and overestimate the success rate of assisted reproductive technologies (4–7). Many also postpone childbearing until ages when the fertility has started to decline (6,8). In Sweden, the mean age of first-time mothers has increased from 24 years in 1973 to 28.5 years in 2013 (9). Older age at conception also increases the risks for adverse maternal and child outcomes (10,11).

In addition to postponed childbearing, a considerable proportion of women and men of reproductive age are overweight or obese and use tobacco and alcohol—which are well-documented risk factors for adverse health, reproductive, and neonatal outcomes (9,12–16). Also, sexually transmitted infections (STIs) can affect fertility, and sexual risk-taking has increased among young adults (17,18). This motivates informing young people about the age-related limitations of fertility and how preconception lifestyle may impact sexual and reproductive health.

Fertility awareness has been defined as ‘the understanding of reproduction, fecundity, fecundability, and related individual risk factors (e.g. advanced age, sexual health factors such as sexually transmitted infections, and life style factors such as smoking, obesity) and non-individual risk factors (e.g. environmental and work place factors); including the awareness of societal and cultural factors affecting options to meet reproductive family planning, as well as family building needs’ (19).

The number of websites addressing preconception health is growing internationally. For example, the web-based fertility awareness tool FertiSTAT.com was launched in 2009 by researchers at Cardiff University in the UK (20). The ‘Your Fertility programme’, funded by the Australian government, launched an interactive website (yourfertility.au) in 2012 that successfully has reached a broad public internationally, including health professionals (21). In 2015, the Flemish Minister of Welfare in Belgium launched a website on preconception care, gezondzwangerworden.be, targeting both professionals and consumers (22). Also, in 2015, the Danish Ministry of Environment and Food and the Ministry of Health launched *MaybeBaby* (maybebaby.dk), a web-based education material on fertility. Moreover, in 2016, the first consumer-focused preconception health website in the US was launched by the National Preconception Health and Care Initiative (PCHHC) (showyourlovetoday.com). The aim of the latter was to improve the health of young adults through guidance and empowerment and by bridging the gap between them and their healthcare providers (3).

In Sweden, Internet sources with evidence-based preconception health information targeting both women and men are limited. The webpage 1177.se has some information on reproduction, but information about the importance of health, age, and lifestyle factors on fertility is limited. Consequently, there is a need for easily accessible, evidence- and web-based information targeting young adults, to improve fertility awareness and encourage reproductive life planning.

The Reproductive Life Plan (RLP) is a non-normative counselling tool, recommended by the Centers for Disease Control and Prevention (CDC), for reflection on intentions and strategies for successful family planning within the context of personal life goals and values (1,23). RLP aims to increase knowledge on fertility and preconception health among young women and men, to enable informed choices about sexual and reproductive health.

The RLP concept has previously been evaluated in contraceptive counselling in Swedish settings. Using RLP increased women’s knowledge about reproduction and folic acid intake (24). It was positively received by clients and well adopted by healthcare providers, who stressed the importance of expanding the RLP usage to other arenas (25,26). As e-health is a growing field of healthcare, developing the RLP concept into a web-based resource that clients could use and healthcare providers could refer to during counselling was therefore suggested (26).

The aim of the present study was to develop and evaluate an evidence-based fertility awareness website, reproduktivlivsplan.se, to increase awareness of fertility and provide guidance for improved preconception health and care among individuals and healthcare providers.

## Materials and methods

### Website development process

#### Content

The textual content of the website was based on two 28-page colour booklets, designed and previously used by our research group when evaluating the RLP tool during contraceptive counselling and STI testing in a randomized controlled trial and a mixed method study (24,25). The booklets contained evidence-based information on reproductive life planning, healthy lifestyle, fertility and reproduction, and a fertility awareness quiz. Most midwives (33 out of 36) thought the booklet was very or rather useful, and it was considered a good conversation starter (25). We also added a selection of relevant references relating to each section of the website. Furthermore, the website is being translated into six languages commonly spoken by minority groups in Sweden: English, Arabic, Spanish, French, Greek, and Somali.

#### Design and layout

We consulted an external web design company to construct the site. The aim was a simple, responsive design with minimal columns and a limited amount of text for easy navigation of the website. For structural consistency, the main heading ‘Reproduktiv Livsplan’ (in English: Reproductive Life Plan) was kept visible on all pages. Images and colours were chosen from a gender-inclusive perspective as well as for thematic and aesthetic harmony. For increased usability, the website was designed to be compatible with mobile devices.

The website menu was divided into six main headings: ‘A plan for the future’, ‘Conception’, ‘Fertility and health’, ‘Test your knowledge’, ‘Your reproductive life plan’, and ‘Do you want to know more?’ Each heading has one or more subheadings covering the impact of age on fertility, preconception risk factors, and health-promoting information. This included a fertility awareness quiz, personal print-and-go guides for reproductive life planning and preconception counselling, as well as links for further information—all geared towards both women and men.

**Figure 1.**
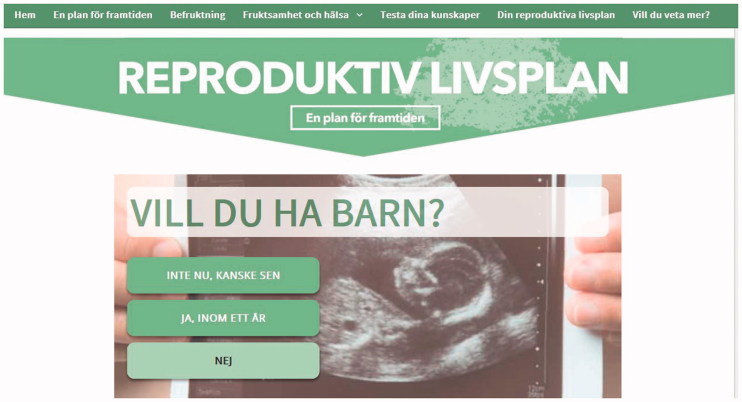
The website ‘Reproduktivlivsplan.se’.

**Figure 2.**
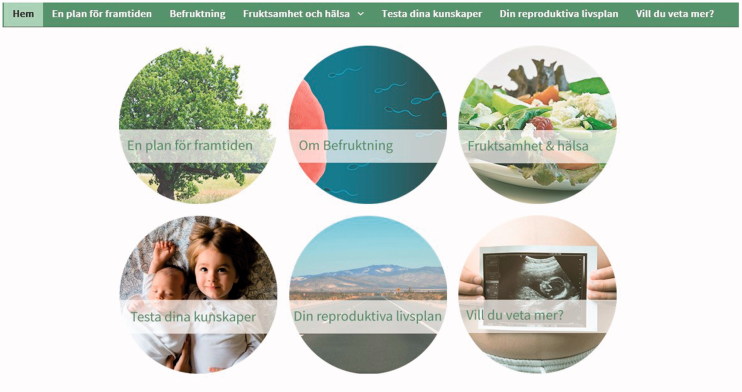
Images displayed and labelled as the menu headings.

To catch the user’s attention, the RLP question ‘Do you wish to have children?’ was displayed in large font on the centre of the screen. The user responds by choosing from three different alternatives: ‘Not now, perhaps later’, ‘Yes, within one year’, or ‘No’. Each response alternative is followed by preconception advice tailored to the chosen response (Figure 1). Below the RLP question, six smaller images were displayed and identically labelled as the menu headings, with short-cut entrances to the different sections (Figure 2).

### Evaluation and data collection

The website was evaluated first by nursing students that represented the primary target group and later by a multidisciplinary expert group of reproduction researchers (the expert group consisted of members of ReproUnion, an EU-funded cross-border collaboration between Sweden and Denmark within the area of reproductive medicine in the Oresund Region, www.reprounion.eu). Finally, after amendments, the website was evaluated by nurse-midwives using the website in contraceptive counselling.

### Target group and group of experts

Nursing students between 20–30 years from two universities in Sweden were invited to participate in focus groups interviews. We chose this convenience sample of nursing students since we presumed that they were similar to the target group of young people between 20–30 years of age. The students received verbal and written information about the study by the course administrator. One moderator led the interviews. Participants were provided with a link to a draft version of the website for online navigation on individual laptops during the interviews. A semi-structured topic guide with open-ended questions was used, in which participants were asked to assess the webpage’s *content*, *design*, and *layout*. Participants were asked to view the front page and share their first impressions, including perceived target group(s) and objective(s). They were further asked to discuss sufficiency, clarity and saturation of information, and trustworthiness of the website source, along with suggestions for further improvements. While navigating on the website, participants provided feedback on acceptability of colours, images, and how they viewed the website in terms of user-friendliness.

The expert group was asked to revise the draft version of the website during a research seminar in December 2016, attended by two of the authors (M.E.R. and T.T.). The website was displayed by the first author and simultaneously discussed by the group during online navigation.

### Healthcare providers

Nurse-midwives (*n* = 37) working with contraceptive counselling at seven clinics in southern Sweden were introduced to the RLP concept and the RLP website by the authors M.E.R. and T.T. during a three-hour presentation and workshop. We then asked the nurse-midwives to use the website over a three-month period (June through August 2017) during contraceptive counselling, and thereafter anonymously complete a short 14-item questionnaire. The questionnaire covered brief and general background data regarding participants’ age, experience of working with contraceptive counselling, and number of weekly contraceptive counselling visits. It also covered participants’ views on information provided on the RLP website, and its usefulness for clients and healthcare providers. Two reminders on returning the questionnaire were sent to the participants.

### Ethical consideration

The study was performed in accordance with the ethics of scientific work as outlined in the Declaration of Helsinki and the national guidelines on research ethics. All participants (students and nurse-midwives) were informed that participation was voluntary, that they could withdraw participation at any time without reason or negative consequences, and that no unauthorised persons would have access to the data. They were also informed that all collected data would be presented on a group level and that no individual could be identified. Students signed an informed consent. The researchers’ contact information was given to participants. According to Swedish law, there was no need for additional approval from a regional ethical committee.

### Data analysis

The focus group interviews were audio recorded, transcribed verbatim, and analysed deductively by manifest content analysis. Collected questionnaire data were analysed descriptively.

## Results

### Focus groups and individual interview

Final-year Swedish-speaking nursing students (3 males and 16 females) aged 20–30 years took part in three focus group interviews, with 4, 5, and 10 participants each. A fourth scheduled focus group interview was transformed into an individual interview because of unexpected participant drop-out. Interview data were collected at each study site between March and May 2016 and between March and April 2017.

### Content

First impressions of the website revealed comments such as: ‘appealing’, ‘lucid’, ‘simple and good’, ‘modern’, ‘clear’, but also comments such as ‘a bit sterile’ and ‘anonymous’. The fertility quiz was highly appreciated. Participants generally found the content of the website to be sufficient and interesting, and it was new to some.

The participants considered the target group to be clear, directed towards both women and men, including individuals with no plans of having children. However, several participants believed it would be difficult to get women and men who did not intend to have future pregnancies to visit the website:

‘After all, I still think this website mainly targets those who want children. […] You enter this page if you want children in your life, now or later, basically.’

[General agreement]

‘And even if it’s great with all these links on where to turn [i.e. for contraceptive advice], I don’t think you’d visit the website if your primary goal was to not have children …’ (Focus group interview 2)

Many liked the brief format of the website and appreciated the external links where interested readers could access further information. Others suggested the website could be even more comprehensive and wanted to have information on a variety of related topics, ranging from in-depth information on contraceptive methods, child rearing, to rules and regulations regarding parental benefits:

‘I have to say, I like the brief format. With limited amount of text, you can follow it easily and concentrate on the actual information. It doesn’t take a whole day to read through it.’

‘Well, yes, I’m not sure. I might be a bit of a nerd but if I buy something, I want to read everything there is to read about it. I don’t have any children of my own, but if I would plan on getting pregnant, I guess I’d like to read everything there is. So, perhaps more text, if you ask me.’

‘I prefer it to be brief.’

‘See, I’d never have the patience to read long sections of text.’

‘You’d probably lose some [readers] with too much information.’

‘Yeah, well then it’s smart to have options, like “would you like to read more … go to …” because, at this website, for this purpose, it’s probably just enough … since it refers to external links for those that are interested in further information.’ (Focus group interview 1)

Some criticized the website’s heteronormative language and called for more focus on challenges faced by the lesbian, gay, bisexual, transgender, and queer (LGBTQ) community, in terms of reproductive health. Accordingly, a researcher in gender studies reviewed and adjusted the text where appropriate.

‘My first impression was that this website was mainly directed towards heterosexual couples. At least at first sight. But, that is mostly pictorially …’ (Focus group interview 2)

‘Since their [transgender people’s] reproduction has been prohibited in a way, I believe it would be good to include them and mention this too.’ (Focus group interview 4)

Most participants found the website to be trustworthy with reliable information from a valid source (Uppsala University). However, there were a few suggestions on clearly identifying the website source and making the university logotype more visible as well as including the contact information, all of which have been amended. In addition, one respondent suggested a chat forum to increase the website’s credibility:

‘For me, having a chat forum makes it seem like a more serious website. Then it’s always updated, in a sense. Someone is in charge … If I pose a question, someone will answer it, which means somebody is responsible for the content. It gives an impression of seriousness and indicates that it is trustworthy.’ (Focus group interview 4)

### Design and layout

There were some remarks about the colours and images, where some participants found the colours a bit too dark. Others voiced that the images looked semi-professional, and some believed they appeared as too heteronormative. Participants suggested using less heteronormative images with stronger coherence with the website’s theme and colours. Some images were replaced and adjusted following participants’ comments.

To test usability, participants were asked to explore the website and test the ease of use while simultaneously sharing verbal feedback. Overall, they found the website to be user-friendly, and information sought for was found swiftly. Participants stated that the website was also easily accessible on mobile devices, and well-structured in its format. There were some suggestions on how to better direct the user from one page to the next by adding ‘next-buttons’ at the bottom of each page. This was later amended. Participants revealed a few dead-end links, which were subsequently corrected.

### Expert group

The expert group suggested the content of the website to focus more on age-related fertility decline considering its importance in connection to fertility problems. Amendments were made according to the suggestions.

### Healthcare providers

Of 37 invited nurse-midwives, 24 filled out and returned the questionnaire, resulting in a 65% response rate. Mean age of participants was 50 years (range 29–64 years), and mean years of experience in contraceptive counselling were 14 years. The participants had on average 10 visits for contraceptive counselling per week (range 3–15). Nurse-midwives’ general views on the RLP website are presented in Table 1. All except two of the nurse-midwives (22 of 24) had visited the website. Reasons for not having visited the website were: ‘lack of time’, ‘not motivated’, or ‘did not know about the website’. The majority (20 of 21) were positive towards using the RLP website during client consultations, but few had done so; seven nurse-midwives had mentioned it to clients, and, out of these, three had also shown the website to clients on 18 occasions, in total. All nurse-midwives intended to use the website in future contraceptive counselling consultations (see Table 1).

**Table 1.** General views of the RLP website among nurse-midwives working with contraceptive counselling (*n* = 22).

Variable	Total number of responses	Number of responses
As a nurse-midwife, I found the RLP website to be …	19	
Very or rather useful		17
Neither useful nor useless		1
Very or rather useless		1
For clients, I found the RLP website to be …	20	
Very or rather useful		18
Neither useful nor useless		2
Very or rather useless		–
My general attitude towards using the RLP website in client consultations was …	21	
Very or rather positive		20
Neither positive nor negative		1
Very or rather negative		–
Clients’ responses to the RLP website were …	22	
Not applicable^a^		11
Very or rather positive		11
Neither positive nor negative		–
Very or rather negative		–
In the future, I will use the RLP website …	21	
Always/often		13
Sometimes		8
Seldom/never		–

a Have not showed/referred to website during client visit.

## Discussion

We developed Reproduktivlivsplan.se to increase awareness among individuals regarding the impact of age and lifestyle in relation to fertility and reproductive health, and to encourage reproductive life planning. The website was positively evaluated by young women and men, and by healthcare providers; thus, it has the potential to provide general guidance supporting healthy lifestyles and to serve as a tool for healthcare providers to increase preconception health awareness.

Proper and reliable preconception health information may ultimately help prevent adverse pregnancy outcomes. Most participants in our study found the information on the website to be interesting, sufficient, and even new. On one hand, it has been argued that knowledge, awareness, and belief in the benefits of preconception care do not necessarily lead to improved preconception health practice (27). On the other hand, without sufficient knowledge, young people will not even have the option of making informed decisions regarding childbearing and sexual and reproductive health.

In Sweden, as in most European countries, information on preconception health available for healthy women and men is still limited. Existing recommendations, guidelines, and web information are often fragmented and inconsistent (28). Consequently, efforts are needed to ensure that correct recommendations are consistently shared by using sufficient and validated strategies. As the Internet was an important source of information for women seeking pregnancy-related information already in 2004 (29), dissemination of web-based health information is suitable to guide preventive actions in the preconception period. Reproduktivlivsplan.se is the first website focusing on reproductive life planning and evidence-based key messages on preconception health in Sweden.

There are many challenges to successfully target young individuals with health information preconceptionally. Many seem to start seeking information once pregnancy is confirmed or when having difficulties conceiving. Informants in our study believed it would be difficult to motivate women and men without (immediate) desire for children to visit the website and to establish a reproductive life plan. Other identified barriers include lack of awareness, perceived sufficient knowledge or not belonging to the target group, and misunderstanding the aims of preconception care (30,31). Still, for young people to benefit from information about preconception health recommendations, information needs to be available early in life—regardless of plans for future childbearing.

Opportunities for offering information on preconception health and lifestyle include contraceptive counselling and screening for STIs and cervical cancer, in which information about fertility preservation plays an important part. In Sweden, contraceptive counselling is free of charge, and nurse-midwives, who carry out 80%–90% of all contraceptive counselling consultations, therefore have an excellent opportunity to spread information on preconception health and care. The website can be used by the healthcare provider *during* client consultations by navigating the website together with the client. Clients can also be encouraged to visit the website *prior to* visits, to be better prepared for discussions of personal interest. Also, other professions, such as educators, are important key persons in disseminating preconception health messages, and the website could be a valuable tool during sexual health education in schools.

Information material is often ethnocentrically biased with cultural and linguistic shortcomings. To address the needs of non-Swedish-speaking people, we decided at a late stage of the website-developing process to translate the website into six languages commonly spoken by immigrants and minority groups in Sweden. Further evaluation is needed for the cultural translation of the messages and for assessing the value of the multilingual approach in these groups.

We evaluated the website using both qualitative and quantitative methods. Focus group interviews create a non-judgemental environment, which can encourage participants to share their views without the need for consensus. Qualitative data enable understanding of the informants’ perspective, which is important for successful implementation of new working procedures in clinical practice. All informants were nursing students, with the majority being females—a group that may have greater knowledge and/or interest about health information and reproduction compared to the general population, including men. Greater diversity among informants regarding gender, educational level, and non-Swedish-speaking people would have added strength to our study, and interviews with men are already ongoing. Compared to individual interviews, focus group interviews might not be as efficient in covering maximum depth on an issue. However, the informants were highly engaged and discussed the website openly, extensively, and from a wide range of perspectives. All interviews, including the individual interview, provided constructive criticism and valuable suggestions to improve the website. Since the informants were only interviewed once, potentially valuable feedback on the modifications of the website was not collected, and this might be considered as a limitation. On the other hand, an expert group gave feedback on the content during the development process, and the final version of the website was positively evaluated by the nurse-midwives.

Despite several reminders, only 24 out of 37 nurse-midwives returned the questionnaire. Also, even though all except one of the nurse-midwives were positive towards the website, only seven had used it during client encounters, with lack of time being given as the most common reason. This could possibly have affected the results. It also demonstrates the challenge of implementing new working procedures, and future studies can evaluate the use of RLP in clinical encounters. Measuring knowledge increase and pregnancy intention before and after contraceptive counselling guided by the RLP webpage would also be of value. To evaluate the use of the website, we are measuring the website traffic regularly, and currently the website has had more than 30,000 views since it was launched in 2017. The work on spreading it to both young individuals and healthcare providers is on-going, e.g. through radio, social media, information meetings with healthcare providers, and at national and international conferences.

The website reproduktivlivsplan.se was well received among users and healthcare providers and may provide individuals with general guidance to achieve their reproductive goals. It has the potential to serve as a tool for healthcare providers in preconception counselling and for educators when discussing sexual and reproductive health.
